# The Role of Food Supplementation in Microcirculation—A Comprehensive Review

**DOI:** 10.3390/biology10070616

**Published:** 2021-07-02

**Authors:** António Raposo, Ariana Saraiva, Fernando Ramos, Conrado Carrascosa, Dele Raheem, Rita Bárbara, Henrique Silva

**Affiliations:** 1 CBIOS (Research Center for Biosciences and Health Technologies), Universidade Lusófona de Humanidades e Tecnologias, Campo Grande 376, 1749-024 Lisboa, Portugal; 2 Department of Animal Pathology and Production, Bromatology and Food Technology, Faculty of Veterinary, Universidad de Las Palmas de Gran Canaria, Trasmontaña s/n, 35413 Arucas, Spain; ariana_23@outlook.pt (A.S.); conrado.carrascosa@ulpgc.es (C.C.); 3 Pharmacy Faculty, University of Coimbra, Azinhaga de Santa Comba, 3000-548 Coimbra, Portugal; framos@ff.uc.pt; 4 REQUIMTE/LAQV, Rua Dom Manuel II, Apartado 55142, 4051-401 Oporto, Portugal; 5 Northern Institute for Environmental and Minority Law (NIEM), Arctic Centre, University of Lapland, 96101 Rovaniemi, Finland; braheem@ulapland.fi; 6 School of Sciences and Health Technologies, Universidade Lusófona de Humanidades e Tecnologias, Av. Campo Grande 376, 1749-024 Lisbon, Portugal; a21705573@alunos.ulht.pt; 7 Research Institute for Medicines (iMed.ULisboa), Faculdade de Farmácia, Universidade de Lisboa, Av. Prof. Gama Pinto, 1649-003 Lisbon, Portugal; 8 Department of Pharmacy, Pharmacology and Health Technologies, Faculdade de Farmácia, Universidade de Lisboa, Av. Prof. Gama Pinto, 1649-003 Lisbon, Portugal

**Keywords:** cardiovascular, food quality, food safety, food supplements, microcirculation, nutrition

## Abstract

**Simple Summary:**

There is increasing evidence showing that the pathophysiology of several forms of cardiovascular disease (CVD) are characterized by microcirculatory dysfunction, which may in fact precede clinical manifestations. For several decades, food supplements have been explored with the aim of restoring microcirculation. Considering the increasing growth of the food supplement industry in recent years and the great impact that CVD has on human health, the present study aimed to provide an up-to-date review of the literature on the role of food supplementation in microcirculation. In this work, the main legal aspects in the European Union and the United States of America concerning food supplements and the beneficial effects that these supplements may have on the population for microcirculation are discussed. Although further studies are needed, especially in humans, there are some data that suggest the beneficial effect of the following supplements: *Ruscus aculeatus* L., *Centella asiatica* L., *Ginkgo biloba* L., *Salvia miltiorrhiza* Bunge, *Crataegus* spp., Ginseng, *Mangifera indica* L., *Aesculus hippocastanum* L., *Hamamelis virginiana* L., and *Vitis vinifera* L.

**Abstract:**

(1) Background: Cardiovascular disease (CVD) is a major public health concern worldwide and a key cause of morbidity and mortality in developed countries. Accumulating evidence shows that several CVD forms are characterized by significant microcirculatory dysfunction, which may both cause and be caused by macrovascular disease, often preceding clinical manifestations by several years. Therefore, interest in exploring food supplements to prevent and restore microcirculation has grown. Given the continuous need to expand the available therapeutic arsenal for CVD, the food supplements market has recently grown and is expected to continue growing. (2) Methods: We provide an authoritative up-to-date comprehensive review of the impact of food supplementation on microcirculation by analyzing the European and American legal food supplements framework and the importance of food safety/food quality in this industry. We review the main literature about food bioactive compounds with a focus on microcirculation and some main food supplements with proven benefits. (3) Results: Despite a lack of scientific evidence, diet and microcirculatory function are clearly connected. The main food supplement examples in the literature with potential beneficial effects on microcirculation are: *Ruscus aculeatus* L., *Centella asiatica* L., *Ginkgo biloba* L., *Salvia miltiorrhiza* Bunge, *Crataegus* spp., Ginseng, *Mangifera indica* L., *Aesculus hippocastanum* L., *Hamamelis virginiana* L., and *Vitis vinifera* L. (4) Conclusions: further clinical trials are necessary to better explore the effects of these food supplements.

## 1. Introduction

### 1.1. Microcirculation in Physiological Conditions

Microcirculation consists of a network of small blood vessels (small arteries, arterioles, capillaries, venules, and small veins) that establish a frontier with the interstitium and with lymphatic vessels, a functionally connected triad that collaborates in maintaining the homeostasis of tissues as well as of the entire cardiovascular system [1]. Small arteries and arterioles ensure the delivery of nutrients and oxygen to tissues with a given perfusion pressure and according to local demand. Tissue nutrition itself occurs at the capillary interstitial interface by a combination of three mechanisms: the filtration of plasma fluid-containing nutrients; gas diffusion; and the pinocytosis of macromolecular nutrients. Waste products are extracted by venules, small veins, and lymphatics, the last of which also prevents the accumulation of interstitial fluid and the development of edema [2].

Microcirculation displays several mechanisms that collaborate in regulating organ perfusion. First, small arteries and arterioles display myogenic activity by which they are able to change the vascular tone in response to changes in blood pressure to guarantee that perfusion remains constant over time. Second, neurotransmitters released from the autonomic nervous system, as well as endocrine and local mediators, especially from the endothelium, also contribute to changing the vascular wall tone of arterioles and venules, which changes both organ perfusion and blood pressure in the short term [3,4,5]. For example, the release of vasoconstrictor mediators decreases the arteriolar caliber, which decreases organ perfusion pressure but raises blood pressure. The release of venoconstrictors, however, decreases the caliber of venules and promotes the venous return to the heart, while lowering organ perfusion pressure. Third, for long-term perfusion regulation, microcirculation can trigger angiogenesis, the generation of new blood vessels from pre-existing ones, by playing an important role in tissue development and repair [6].

Considering the important interface it establishes with the tissue interstitium, microcirculation also contributes to local tissue immunity by allowing the adhesion and infiltration of immune cells [7]. Finally, given its small caliber, microcirculation is a favorable site for the accumulation of hemostatic plugs. Under physiological conditions, the microvascular endothelium expresses a low level of adhesion molecules that limits the adhesion of leucocytes and platelets by preventing inflammation and the occurrence of thrombotic phenomena.

### 1.2. The Role of Microcirculation in Pathological Conditions

Several cardiac, vascular, renal, metabolic, and even neuropsychiatric conditions have been linked with microvascular dysfunction. Recently, evidence has accumulated suggesting that the earliest manifestations of cardiovascular disease (CVD) occur at the microcirculation level, with macrovascular dysfunction and target organ damage later ensuing [8,9,10,11]. Therefore, therapies that aim to prevent or restore microcirculation have been increasingly explored in recent years [1,12,13].

The role of microcirculation in pathological conditions may be fundamentally reversed compared to physiological conditions, most strikingly with regard to the endothelium. Endothelial dysfunction is, therefore, characterized by: (1) the decreased ability to synthesize mediators with “vasoprotective” actions, especially nitric oxide (NO, i.e., vasodilator, anti-inflammatory) under both resting conditions and stress [14], and the increased synthesis of “vasodisruptive“ (vasoconstrictor, proinflammatory, proaggregator, profibrotic) mediators, especially endothelin-1 [15]; and (2) the increased expression of adhesion molecules in the endothelium, which increases the ability of leucocytes and platelets to adhere to the vascular wall [7]. In addition, myogenic activity may be disrupted or absent in pathological conditions, which prevents adequate perfusion regulation [14]. Consequently, microvascular dysfunction increases the risk of fibrosis of the vascular wall, vascular and tissue inflammation, and ischemic and thrombotic phenomena [1].

### 1.3. Food Supplementation

Food supplements are manufactured from food, isolated nutrients, or food-like compounds, which occur as powders, pills, potions, and other types of medication that are not commonly associated with food [16]. Due to the increasing interest shown in personal health, aging demographics, and successful personalized care products, the demand for food supplements has grown and is expected to continue [17].

Cardiovascular disease is a significant public health concern worldwide and a leading cause of morbidity and mortality in developed nations [18,19]. In 2015, nearly one third of all deaths worldwide were caused by CVD, according to the World Health Organization [20]. Thus the effects of cardiovascular risk and CVD on most food supplements have long since been investigated [17,21,22,23].

Recent research works by Khan et al. (2019) [23] show that food supplements and dietary interventions are beneficial for mortality and cardiovascular outcomes, reporting data that support low salt consumption, omega-3 (ω-3) polyunsaturated fatty acids (PUFAs), and folate supplementation to minimize the risk of CVD. Likewise, calcium and vitamin D combined indicate an elevated risk of stroke. Survival or cardiovascular benefits have not been correlated with other vitamins, minerals, food supplements, and dietary interventions. Vazquez et al. (2019) indicate that the regular ingestion of probiotics, which maintain the equilibrium of intestinal microbiota, can have cardiovascular benefits, at least in part, because of their potential to minimize oxidative stress [22].

A very recent study by Baumgartner et al. (2020) concludes that better lipid regulation can be accomplished through food supplementation with demonstrated effectiveness at plasma low-density lipoprotein cholesterol levels. It is possible to apply these items to preventive strategies with all subjects (universal prevention) or with a view to concentrate more on patients with risk factors and/or CVD (care-related prevention) [21].

Considering these premises, the aim of this study is to provide an objective and up-to-date comprehensive review of the impact that food supplementation may have on microcirculation. In addition, given the overwhelming number of studies supporting the vast benefits of a vegetable-based diet and considering the increasing global awareness for ecological sustainability as well [24,25], only bioactive compounds and food supplements derived from vegetables were included in this review.

## 2. The Importance of Food Safety and Food Quality in the Food Supplement Industry

Undoubtedly, the consumption of dietetic foods, food supplements, and fortified foods has disproportionately increased in the last 20 years in all age groups in developed countries, the United States of America (USA) and Europe, and the Asian Market. The global food supplements market has increased by around 120 billion dollars with annual growth in recent years at approximately 6% [26], especially for those whose main ingredients are plants, based on the common idea that they are natural products and pose no risks to human health [27]. They are known in Europe as food supplements. In the USA, the term dietary supplements is employed to convey this meaning. Nevertheless, this profitable business has provoked the appearance of a public health problem caused by an increase in dangerous relations with a high concentration of supplements, unauthorized composition, or a possible interaction with other supplements and medications [28]. Present European legislation has been criticized for being ineffective and unconvincing by having been relaxed and not being able to offer legal coverage to all new labeled products in the category of dietetic foods, food supplements, and fortified foods [29], despite this legislation stating that information about food derivation and origin must be unblemished and unquestionable for consumers [29].

The European Union (EU) considers these products to be food, and they are regulated by European food law (Regulation (EC) 178/2002 and Directive, 2002/46/EC) [30,31]. However, the USA has specific regulations about them [32]. Current EC Regulation has been modified by the following regulations and directives to include additional substances: Commission Regulation (EU) 2017/1203, Commission Regulation (EU) 2015/414, Commission Regulation (EU) No 119/2014, Commission Regulation (EU) No 1161/2011, Commission Regulation (EC) No 1170/2009, and Commission Directive 2006/37/EC.

Moreover, product legislation is not fully harmonized in the European Single Market as food supplements are defined as food products whose purpose is to supplement normal diet and that consist of concentrated sources of nutrients or other substances with a nutritional or physiological effect [33]. Nevertheless, the indiscriminate use of different terms, such as “food/dietary supplements” and “nutraceutical”, may be controversial as no unified legislation across countries exists. In some countries, the term nutraceutical is almost equivalent to food/dietary supplements/products. Furthermore, food products, food additives, and even drugs are sometimes marketed as nutraceuticals [34].

All EU Member States have their own Food Security Agency, which monitors the food supplements trade and provides consumers with information and advice about registrations, the selling of products, suppliers, labeling, and legal requirements (French Food Safety Agency (AFSSA, Agence Française de Sécurité Sanitaire des Aliments); Food Standards Agency (UK), 2018; Bundesinstitut für Risikobewetung (BFR), (Germany), 2018; Spanish Agency for Food Safety and Nutrition (AESAN), 2018 [33,35,36,37]). Nevertheless, AESAN has developed an appropriate program against illegal food supplements trade [33]. In any case, the first step for warranting product safety and quality is to effectively differentiate between products with similar definitions and regulations, such as food preparations for special nutrition, foods intended for infants, and medicinal products.

The definition of over the counter (OTC) is linked with the product type as this classification does not require medical prescriptions, unlike medicines. The OTC sales channel is limited to the pharmacy office in Spain. Nevertheless, other European countries and the USA are also open to parapharmacies, supermarket corners, and drugstores [38]. The marketing strategy correlates with the regulations for the corresponding product category. The registration of food supplements is easier and much quicker than the registration of medicines. As a result, there is a huge difference in the marketing strategy of food supplements and OTC medicines [39]. For this reason, fraud can appear more with OTC products. Hachem et al. (2016) analyzed food supplements marketed by weight and mainly purchased on the Internet. They analyzed 164 samples, and only 44% were truly natural or had a composition that actually matched their label. Moreover, the presence of certain adulterants in some product combinations could be considered dangerous for health [26].

Food supplements should be taken at the recommended daily doses indicated on the label product, and this use recommendation must never be exceeded and cannot be a substitute for a balanced daily diet. Its labeling must clearly facilitate the identification of its components and facilitate official controls through its registration in the food supplements database [33]. Noncompliance comes about from not complying with the applicable regulations or, after evaluating the available information about the product, from concluding that it is not safe or does not provide sufficient safety guarantees as a food supplement for consumers. For these purposes, the provisions of the “Guide for the official control of labeling and composition of Food Supplements” can be followed [33].

In more detail, noncompliance may be considered, among other factors, to be [33]:

- A food supplement for sale on the market that has not been previously noted or included in the corresponding food supplements list or does not coincide with what has been noted and commercialized;
- The food supplement labeling does not comply with the provisions of the general and specific regulations for labeling food supplements;
- The labeling declares a nonharmonized substance and does not justify prior marketing in another Member State;
- The labeling of the food supplement includes unauthorized harmonized substances or substances in larger quantities than those authorized in those cases for which a maximum limit has been established.

For consumers, product quality means its suitability for use, reliability, efficacy, and, above all, safety. Elements that may affect the safety of plant food supplements (PFS) are [40]:

- The presence of toxic compounds;
- The presence of pharmacologically active substances;
- The presence of addictive or psychotropic substances;
- Adverse reactions to, and drug interactions with, otherwise nontoxic substances;
- Genetic variants among plant species;
- Differences in processing and manufacturing conditions. Some other problems are addressed in this section;
- Misidentification of the initial plant source;
- Adulteration by other plants;
- Environmental contamination (e.g., with heavy metals and pesticide or herbicide residues);
- Biological contamination (mycotoxins, microorganisms);
- Addition of illegal substances.

In order to control this international trade, the EU has established controls at the borders of Member States to prevent the entry of products at high risk of carrying some type of hazard, because food supplements form a part of those with a high percentage of irregularities. To this end, in 1970, the EU created the Rapid Alert System for Food and Feed (RASFF), which is a mechanism that ensures the flow of information to enable swift reactions when public health risks are detected in the food chain. In this way, the detected information can be shared efficiently by members [41].

RASFF notifications usually report the risks identified in food, feed, or food contact materials in the market in the notifying country or detained at an EU point of entry. The notifying country reports the risks it has identified, the product and its traceability, and the measures it has taken [42].

The RASFF list has registered 64,413 food notifications from 1 January 2015 to 15 February 2021, of which 1465 notifications belong to the products category “dietetic foods, food supplements, fortified foods”. This brings the total exchanges in RASFF in 2019 to 14,803 notifications, once again a number that has never been higher. Thus, the most notified product category is “dietetic food, food supplements and fortified foods” (187, 1.26% notifications), while “fruit and vegetables” (174, 1.17%) has reached second place over “meat and meat products” (137, 0.92%) [42].

**Figure 1:**
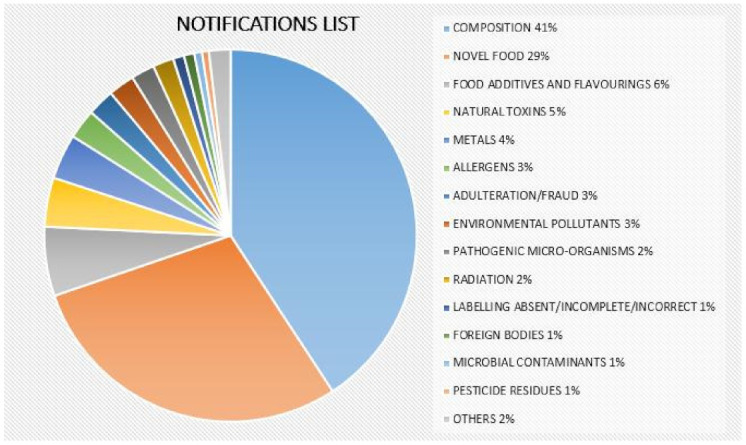
The range of RASFF notifications in the “dietetic foods, food supplements, fortified foods” category from January 2015 to February 2021.

The range of notifications from 1 January 2015 to 15 February 2021 in this product category is shown in Figure 1. It should be noted that the RASFF Portal establishes 40 different product category foods, and Figure 1 only includes the 14 largest categories. There are 106 notifications for the unauthorized novel food ingredient cannabidiol (tetrahydrocannabinol is a psychotropic substance that is naturally present in cannabis plants and is not allowed in food supplements) in either oil drops or food supplements, 76 notifications for high contents of vitamin A, B, or D in food supplements, and 65 notifications for high contents of metals (magnesium, mercury, lead, zinc, nickel).

**Figure 2:**
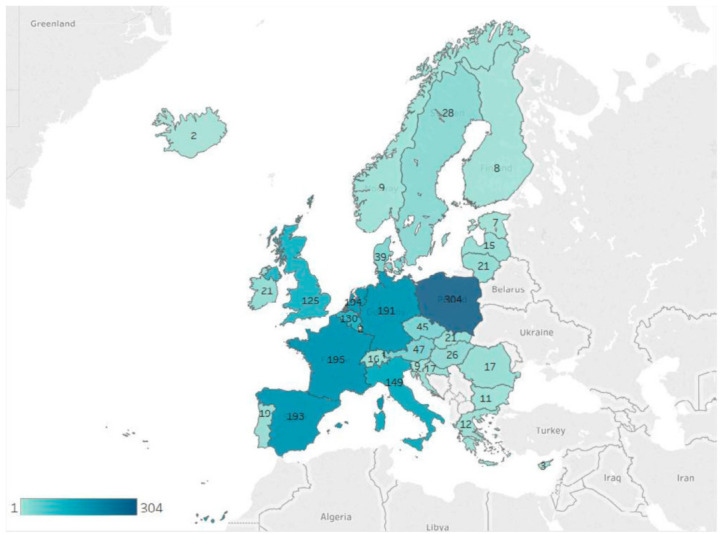
RASFF notifications by RASFF member countries, identified as the origin of the notified product expressed as number of notifications per country of origin in 2019 [42].

Regarding notifications for origins from nonmember countries in 2019, there were three countries at the top of the RASFF list: China with 379 notifications, Turkey with 330, and the USA with 219 [42] (Figure 2).

Several irregular situations of food supplements have been collected by different authors, such as the supplements themselves (e.g., interbatch variation, contamination, and potential drug interaction) [27], weak links in regulations and challenges to enforcement [43,44], mislabeled, contaminated, and adulterated supplements [29], and evaluations of purity and dose [32].

These data provide us with an idea of the magnitude of the problem and the enormous risk to which consumers are subjected, given the lack of food safety and quality of these products. In spite of border line controls, a large number of supplements are traded via electronic commerce, often by companies registered outside the EU, and reach consumers via the postal service, where goods are not subjected to the same rigorous safety checks [28]. Some examples of the most sold food supplements on the Internet are protein powder, calcium supplements, vitamin E, and slimming products. Currently, there is a boom in ecommerce websites, where Chinese consumers can order foreign products online. Other widely accepted products by consumers are erectile dysfunction (ED) supplements, which are featured in the online marketplace on pages with claims that they naturally treat ED. However, their efficacy and safety are largely unknown, which limits the ability to counsel patients regarding their use [44]. In a previous study, the authors identified 19 unique ingredients in testosterone-boosting supplements, and the literature review revealed 191 studies involving the 10 most frequent ingredients with different properties and efficacies.

Interestingly, the RASFF registered 84 notifications of products with sildenafil and 36 with tadalafil (2015 to 2020). Both have effects on ED. The lack of safety insight has been counteracted by numerous studies showing different results concerning the positive effects of food supplements and quality products traded in Europe, including testosterone-boosting supplements (T-Boosters) [45], metal in preparations of spirulina tablets [46], vitamin D [47], ginseng herbal medicine control and authentication [48], cottonseed oil and cottonseed meal supplementation [49], supplemental lycopene on the cardiovascular risk factor [50], and propolis as an antioxidant and antimicrobial agent [51].

## 3. European and American Legal Framework of Food Supplements

There are different ways of classifying claims for food supplements in the EU and the USA. In the EU, the three major categories are defined as “nutrition claims”, “health claims”, and “reduction of disease risk claims” [52]. In the USA, there are also three major categories, namely “nutrition content claims”, “structure/function claims”, and “health claims” [26]. There is neither a consensus about nor an overlap between the two different classifications. Indeed, for the scope of this paper, it seemed adequate to consider only two types of claims, “nutritional claims” (which correspond to the EU “nutrition claims”, plus the “nutrition content claims” and “structure/function claims” in the USA) and “health claims” (which encompass the USA “health claims”, and “health claims” plus the “reduction of disease risk claims” in the EU).

Regarding the main subject of this paper, it is clear that we must only focus on “health claims”, which, in this context, comprises two main parts: (1) a substance (regardless of it being food, a food component, or dietary ingredient); (2) a disease- or health-related condition [38]. However, health claim categories differ in the EU and the USA.

In the EU, “health claims” can be divided into three main subcategories: the so-called “function health claims”, the so-called “risk reduction claims”, and the health “claims referring to children’s development” [52].

In the USA, health claims can be generically classified into “authorized health claims” and “qualified health claims”. Authorized health claims, approved by the FDA, must comply with the so-called Significant Scientific Agreement (SSA health claims) or the Food and Drug Administration Modernization Act (FDAMA health claims). However, only SSA health claims are allowed on food supplement labels [38]. When a food supplement does not fully satisfy the SSA but is still recognized for some scientific evidence that can support its intended claim, the FDA may recognize that claim as a “qualified health claim”. Thus, it is worth knowing which food supplements may have beneficial effects on microcirculation by considering that the health claims approved by the EU, the USA, or other governments positively affect consumer choices.

Although it was impossible to find any health claims directly identified with the word microcirculation in the list of claims approved by the FDA [38], some of these claims may, in fact, have direct and indirect implications for microcirculation (Table 1).

Regarding the EU [53,54,55] and despite a multiplicity of authorized claims with direct and indirect implications related to the subject of this paper, which can be generically considered similar to those recognized by the FDA, there are five claims with specific references to the word microcirculation that were not authorized given the EFSA’s previous assessment (Table 2).

Nevertheless, some EU Member States, as well as other countries around the world, allow the use of some substances that are also approved as medicines. For instance, for the microcirculation subject, diosmin is the main constituent of many food supplements. However, depending on the dose, diosmin is also an active pharmaceutical ingredient (API), which means that some issues might occur with these borderline products that can be on the market in line with different legislations [61]. Consequently, the interface between food and pharma needs to be better explained to protect consumer rights and to prevent so-called food medicine-related diseases [62].

The regulatory discrepancies between the main EU and USA markets, to which other large markets can be added such as China [63], Japan [64], Australia [65], or Canada [66], surely justify that harmonizing food supplements’ regulation worldwide could be carried out, similar to, for example, that which already exists in the regulation of human and veterinary medicines for quality, safety, and efficacy parameters.

Additionally, last but not least, an international harmonization in food supplements’ regulation could lead to better marketing conditions globally with a high safety level, because consumers would be provided with better health-related information [67].

## 4. Food Supplements with Beneficial Effects on Microcirculation

There is limited evidence for the influence of diet on the microvascular function [68]. It has been shown that high dietary salt levels and low levels of copper and vitamins C, D, and E are detrimental to microvascular function, which can be prevented by restriction and supplementation, respectively [69,70,71,72,73]. Dietary habits characterized by the consumption of foodstuffs rich in polyphenols, such as anthocyanins and isoflavones, seem to have either direct or indirect beneficial microvascular effects in both healthy subjects and CVD subjects [74,75]. Even though fish rich in omega-3 (ω-3) polyunsaturated fatty acids (PUFAs) has a proven positive effect on microcirculation, dietary supplementation with high fish oil doses has led to negative cardiovascular effects.

In this section, the main vegetable-derived food supplements in which beneficial effects on microcirculation have been demonstrated are discussed, together with their most important bioactive compounds. Supplements derived from animal sources, those consisting in single pharmacological substances, and those with referenced benefits on hemostasis and hemorheology are beyond the scope of this review.

### 4.1. Ruscus aculeatus

**Figure 3:**
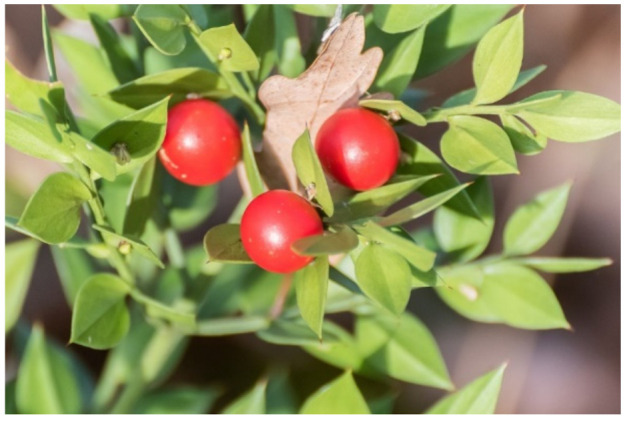
*Ruscus aculeatus* L. photo.

*Ruscus aculeatus* L. (Asparagaceae) (Figure 3), also known as Butcher’s broom is a low evergreen shrub and features in many dietary supplement patents. Its root is used as a phytotherapeutic product, even though its aerial parts are edible [76]. The bioactive compounds identified in the *Ruscus aculeatus* L. extracts include saponins (ruskogenin, neuroruskogenin, ruscin, ruscoside), flavonoids, sterols (sitosterol, stigmasterol, kempesterol), tyramine, coumarin, triterpens, lignoceric acid, glycolic acid, and benzofuranes [56]. The beneficial effects of *Ruscus aculeatus* L. on microcirculation include its endothelial-protecting and venotonic activities.

An extract of *Ruscus aculeatus* L. has demonstrated endothelial-protecting activity due to its antioxidant and anti-inflammatory effects, which prevent leucocyte adhesion and leakage into tissues [77].

The venotonic activity of *Ruscus aculeatus* L. has been reported for several years in both animals and humans. In the hamster cheek pouch model, *Ruscus aculeatus* L. extracts administered either systemically or orally increased venular tone, while maintaining both arteriolar tone and mean blood pressure [77,78]. It appears that the venotonic activity is due to the stimulation of a norepinephrine release from the postganglionic terminals and the activation of alpha-1/2 adrenergic receptors in venular/venous smooth muscle [76,79]. The endothelium is apparently of no particular relevance for this venotonic activity [80]. This activity means that *Ruscus aculeatus* L. considerably benefits patients with venous diseases, including peripheral venous disease (PVD) and hemorrhoidal disease. An extract of *Ruscus aculeatus* L. has been found to significantly decrease the diameter of the popliteal and femoral veins in patients with primary varicose veins in the standing position after 2 h and 1 week, respectively, even though no such result has been observed while supine, and blood flow velocity was significantly quicker [80]. Patients with PVD presenting with the main symptoms of leg edema and the sensation of heavy legs reported they had ameliorated after taking *Ruscus aculeatus* L. extract [81].

*Ruscus aculeatus* L. also has an effect on lymphatic circulation. When administered intravenously to anesthetized dogs, an extract was able to increase the lymphatic osmotic pressure. This means that it was able to extract proteins from interstitial fluid and to enhance lymphatic drainage [82].

Adverse effects related to *Ruscus aculeatus* L. intake are scarce [83]. One exception is a case report that identified an unusual case of *Ruscus aculeatus* L. precipitating diabetic ketoacidosis in a 39 year-old female patient [84].

### 4.2. Hawthorn

**Figure 4:**
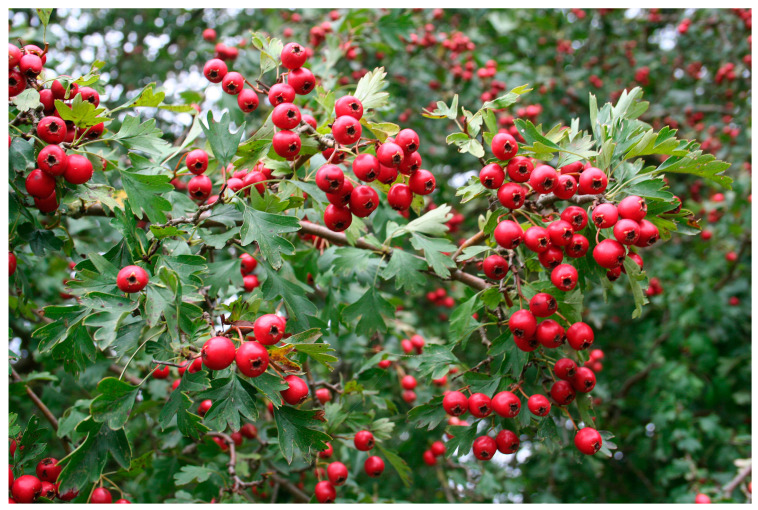
Hawthorn (*Crataegus oxyacantha* L.) photo.

*Crataegus* spp. (Rosaceae) (Figure 4), also known as Hawthorn, is one of the oldest plants used in the Western world for medicinal purposes. Traditionally, only the fruit (i.e., berries) of this plant was used, and then the flowers, seeds, and leaves were later incorporated into the phytotherapeutic arsenal [85]. These botanical parts are phytochemically similar in compositional terms and contain mainly flavonoids (e.g., procyanidins), and only the ratios of these classes differ. Its fruit is rich in hyperoside, while its leaves and flowers are rich in vitexin-2-rhamnoside. The leaves, conversely, are richer in oligomeric procyanidins [65]. In addition, it provides triterpenic (ursolic, oleanolic, and crataegolic) and phenolic (chlorogenic and caffeic) acids [86,87].

Among the several extracts used in herbal products, the WS 1442 hydroalcoholic special extract from the leaves and flowers of *Crataegus monogyna/laevigata* is the most referenced, with its vasorelaxing and endothelial-protecting activities. Regarding the ability to protect the endothelium, WS 1442 inhibits the calcium/protein kinase C/Rho A pathway, which destabilizes the endothelial barrier, while activating the barrier-stabilizing cAMP/Epac1/Rap1 pathway [88]. This extract also modulates the cytosolic calcium concentration in endothelial cells. Interestingly, when acting in isolation, it increases the cytosolic concentration by inhibiting sarcoplasmic endoplasmatic reticulum calcium ATPase (SERCA) transporters and membrane calcium extruders, while activating the inositol-1,4,5-biphosphate (IP_3_) pathway. However, it prevents cytosolic concentrations from rising when endothelial cells are exposed to destabilizing agents, such as thrombin or histamine [89].

Regarding vasorelaxation activity, WS 1442 potentiates NO secretion from the endothelium by activating Src/PI3-kinase/Akt-dependent phosphorylation, which results in endothelial nitric oxide synthase (eNOS) phosphorylation [90]. Procyanidines from *Crataegus oxyacantha* L. and *Crataegus monogyna* Jacq. have also been shown to open BK_Ca_ channels in vascular smooth muscle cells to promote hyperpolarization and, consequently, vasorelaxation [91]. Third, WS 1442 activates nitric oxide synthase specific to red blood cells (rbcNOS) [92], which may also justify this vasorelaxation activity. Finally, it has also been hypothesized that WS 1442 inhibits the angiotensin-converting enzyme [93]. These activities seem to underlie other results, showing that WS 1442 protects against ischemia/reperfusion (I/R) injury in the myocardium as well as consequent arrhythmias [94,95,96].

The most frequent adverse events associated with hawthorn intake are dizziness, vertigo, gastrointestinal complaints, headache, nausea, migraine, palpitations, hypotension, and sedation [97,98]. Acute toxicity symptoms include bradycardia and respiratory depression, which can progress to cardiac arrest and respiratory paralysis. Hawthorn should not be taken while pregnant or during lactation because it decreases the tone and motility of the myometrium [99,100,101].

### 4.3. Centella asiatica

**Figure 5:**
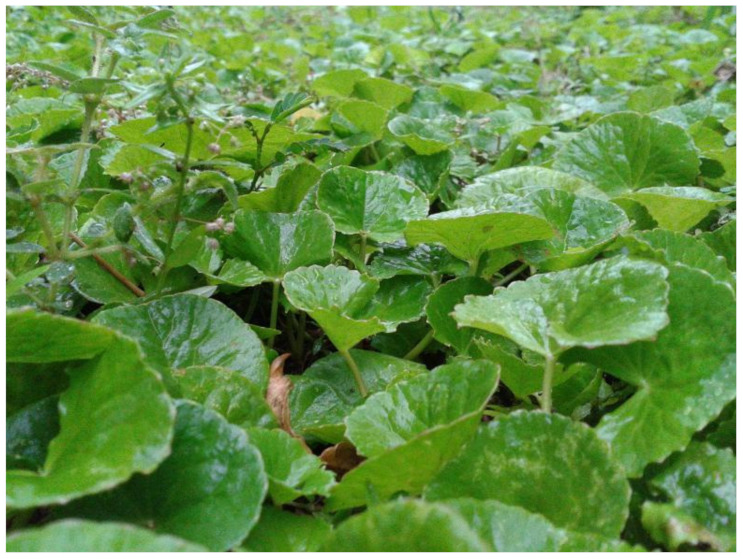
*Centella asiatica* L. photo.

*Centella asiatica* L. (Apiaceae) (Figure 5), also known as “Gotu Kola” or Indian pennywort, is an herbaceous and frost tender perennial plant. It is native to Southeast Asian countries, such as India, Sri Lanka, China, Indonesia, and Malaysia, South Africa, and Eastern Europe. The most relevant bioactive compounds occur in the plant leaves, stems, and roots, and include alkaloids (e.g., hydrochotine), terpenes, triterpenoid saponins (e.g., asiaticoside, asiatic acid, madecassoside, and madecassic acid), flavonoids (derivatives of quercetin and kaempferol), tannins, sterols, and other phenolic compounds [102].

The beneficial effects of *Centella asiatica* on microcirculation are attributed to the triterpenic fraction, which appears to promote collagen synthesis in vascular walls, particularly in venules/veins. When administered to patients with venous hypertensive microangiopathy, this triterpenic fraction decreases capillary filtration and edema, probably by increasing peripheral venous pressure and promoting venous return [103,104].

These triterpenoids also display antioxidant activity, namely on the endothelium [105], which, together with their antiplatelet activity, contributes to protecting against I/R injury, for example, in the brain [106].

Except for a few cases of contact dermatitis, *Centella asiatica* has no known significant adverse effects [107,108]. Animal studies have found that *Centella asiatica* extracts have antispermogenic and antifertility effects on the reproductive system of male rats [109], but are nevertheless safe. However, hepatotoxicity has been reported in both an animal study [110] and a case report. The latter referred to three women who developed jaundice after taking the plant for 20, 30, and 60 days, after which they were clinically diagnosed with granulomatous hepatitis, but their symptoms subsided when they stopped taking the plant [111].

### 4.4. Ginseng

**Figure 6:**
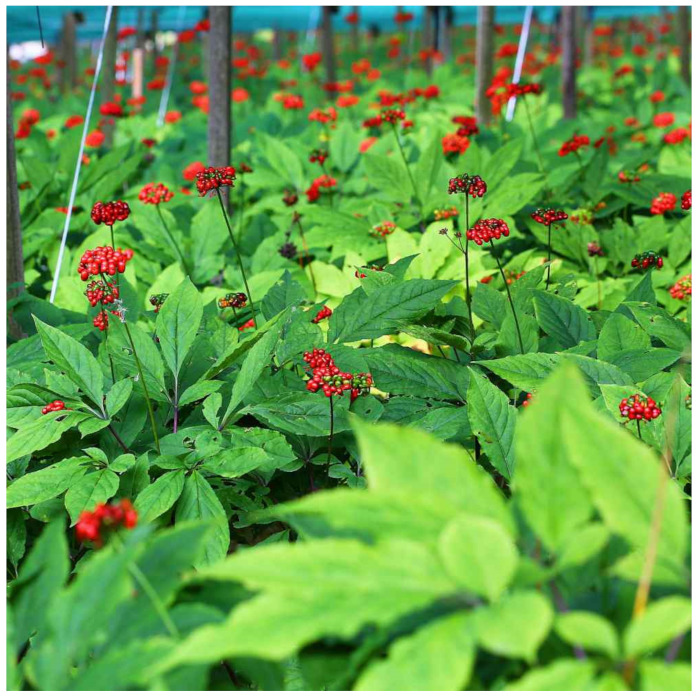
*Panax ginseng* C.A. Meyer photo.

Ginseng consists of different species, namely Korean ginseng (*Panax ginseng* C.A. Meyer) (Figure 6), notoginseng (*Panax notoginseng* (Burkhill) F. H. Chen), and American ginseng (*Panax quinquefolius* L.) [112]. Korean ginseng is a traditional herbal medicine that has been used clinically for over 2000 years with several beneficial effects. Its chemical composition consists of saponins (i.e., ginsenosides Rb1, Rg1, Rg3, Re, and Rd), alkaloids, and phenolic acids [112]. The main actions on microcirculation include vasodilation, endothelial protection, anti-inflammation, and modulation of angiogenesis, attributed to ginsenosides.

Several studies have reported vasodilator activity for extracts of distinct ginseng species in different vascular beds, namely the cerebral circulation. In rats, a crude saponin fraction of *Panax ginseng* C.A. Meyer is able to increase cerebral perfusion [113], attributed to ginsenosides Rb1 and Rg1, which are known to possess strong vasodilator activity in cerebral circulation [114]. Pretreatment with *Panax ginseng* C.A. Meyer saponins reduces auditory damage to the cochlea in guinea pigs, gerbils, and mice [115,116,117], the effects of which may in part be due to a protective role towards cochlear microcirculation by ginsenoside Rb1 [97].

*Panax notoginseng* (Burkhill) F. H. Chen also improves perfusion, with the total saponins attenuating I/R injury in different organs, namely the brain [118,119], and it also protects against oxidative stress, diminishes inflammation, and decreases the expression of caspase enzymes, thereby attenuating the neurological deficit [119,120].

*Panax notoginseng* (Burkhill) F. H. Chen also improves microvascular dysfunction under inflammatory conditions. Upon lipopolyssacharide action in the rat mesentery, the saponin fraction decreases vascular leakage, leucocyte adhesion, mast cell degranulation, and cytokine production [121,122]. Saponosides from *Panax ginseng* C.A. Meyer are also known to protect against homocysteinemia-mediated endothelial and vasomotor dysfunction [123], again due to the effect of ginsenoside Rb1 [124,125].

Several references to the effects of ginseng on the angiogenesis process appear, albeit, to be opposite, which have been attributed to the concentration of different species. *Panax ginseng* C.A. Meyer has antitumoral effects in several models [126], and *Panax quinquefolius* L. possesses a synergistic effect with chemotherapeutic agents for breast cancer [127]. *Panax ginseng* C.A. Meyer inhibits angiogenesis in the adipose tissue of genetically-induced (db/db) or high-fat-induced (C57BL/6J) diabetic mice by reducing the expression of proangiogenic factors such as the vascular endothelium growth factor (VEGF) and fibroblast growth factor-2 (FGF-2) [128,129]. *Panax quinquefolius* L. inhibits the proliferation of the vascular smooth cells in rat aortae when stimulated by several proangiogenic factors, such as angiotensin II, insulin, platelet-derived growth factor, and fetal bovine serum, which is attributed to JAK/STAT pathway suppression [130]. However, *Panax notoginseng* (Burkhill) F. H. Chen is able to promote the proliferation of vascular endothelial cells and the secretion of the VEGF [131]. Administering *Panax ginseng* C.A. Meyer for 8 months to patients with postmyocardial infarction has been found to improve coronary reserve perfusion due to proangiogenic action. It increased the number of angiogenic cells in circulation, while decreasing inflammatory cytokines [132]. This has been tentatively explained by the high concentration of ginsenoside Rb1, which exhibits anti-angiogenic effects both in vitro and in vivo [133]. In contrast, *Panax notoginseng* (Burkhill) F. H. Chen has been shown to promote wound healing and improve microcirculation [134]. The reason for this seems to be the high concentration of ginsenoside Rg1, which also inhibits angiogenesis [133].

Adverse effects associated with ginseng include nausea, diarrhea, euphoria, insomnia, headaches, hypertension, hypotension, mastalgia, and vaginal bleeding [135,136,137]. Ginseng is generally well tolerated, and its adverse effects are mild and reversible [137].

### 4.5. Aesculus hippocastanum *L.*

**Figure 7:**
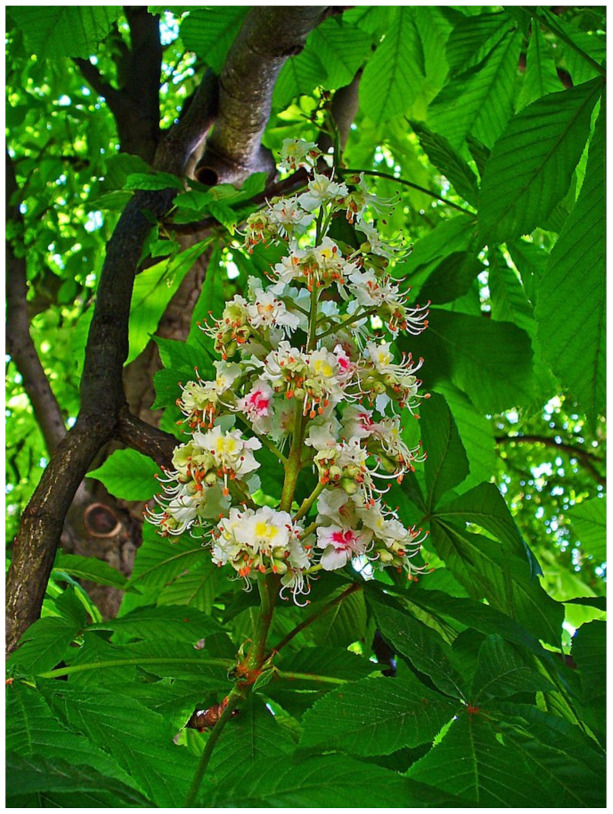
*Aesculus hippocastanum* L. photo.

*Aesculus hippocastanum* L. (Sapindaceae) (Figure 7), also known as Horse Chestnut, is a tree native to southeastern Europe, although it is currently cultivated worldwide. Its fruits contain seeds that resemble sweet chestnuts, although with a bitter taste, and are composed of saponins and flavonoids. The relevance of *Aesculus hippocastanum* L. to vasculature is due to its venotonic, anti-inflammatory, and endothelial-protecting activities, which have been employed for the treatment of venous insufficiency, including PVD and hemorrhoidal disease [138].

One of the main constituents of *Aesculus hippocastanum* L. seed extract is escin, a natural mixture of triterpene saponins [139]. It protects the endothelium against the hypoxia-induced reduction of cellular ATP content, preventing the release of prostaglandins and chemotactic factors, thereby protecting against increased leucocyte adhesion, stasis, and edema [140,141]. However, escin is able to enhance the secretion of prostaglandin F_2_ (PGF_2_), which is known to inhibit the catabolism of mucopolysaccharides in the venous walls and to antagonize the vasodilatory action of histamine and serotonin [142,143]. Therefore, by enhancing the secretion of PGF_2_, it improves the venous contractility and increases the peripheral venous pressure while preventing edema formation [144]. Furthermore, escin also inhibits the activity of several enzymes involved in defining the integrity of the extravascular matrix, namely hyaluronidase, collagenase, elastase, and β-glucuronidase. Therefore, by this enzyme inhibition activity, escin decreases vascular leakage and prevents edema formation [145].

These effects justify the efficacy of *Aesculus hippocastanum* L. in the treatment of PVD. Patients undergoing long-term treatment (oral and/or topical) with escin showed a significant improvement with regard to skin discoloration, pain, edema, and the sensation of leg heaviness [146,147]. According to a systematic review, *Aesculus hippocastanum* L. showed similar efficacy to compression stockings in symptoms of PVD patients [148]. Similarly, patients with hemorrhoids taking *Aesculus hippocastanum* L. showed an improvement in several symptoms, namely burning, itching, and bleeding [149]. Finally, an escin-based topical formulation showed higher efficacy than standard treatment in the improvement of skin microcirculation in diabetic patients with microangiopathy, which justified the significant decrease in the number of ulcerations [150].

Although *Aesculus hippocastanum* L. is generally well tolerated, there are several known adverse effects, including gastrointestinal complaints, headache, vertigo, itching, and allergic reactions [151].

### 4.6. Hamamelis virginiana *L.*

**Figure 8:**
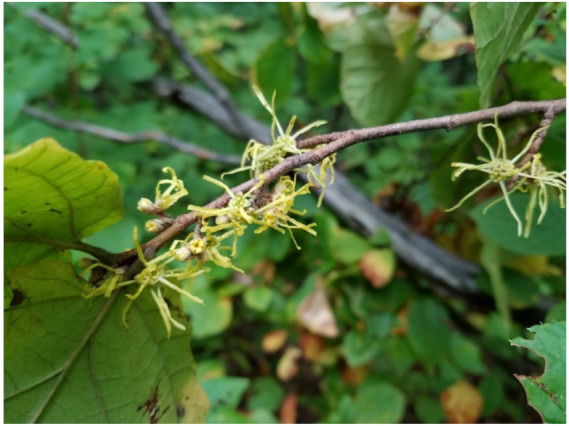
*Hamamelis virginiana* L. photo.

*Hamamelis virginiana* L. (Hamamelidaceae) (Figure 8), also known as witch hazel, is a plant which grows in the northeastern region of the North American continent. It shows important astringent, anti-inflammatory, and local hemostatic effects, which justify its ancestral tradition for the treatment of skin and mucosal diseases, such as hemorrhoids, PVD, and dermatitis [152,153,154]. The leaves and bark of *Hamamelis virginiana* L. are used for therapeutic purposes, and their extracts are composed of tannins, gallic acid, flavonoids (e.g., catechins, proanthocyanins), saponins, and essential oils. Flavonoids and tannins are the main bioactive compounds with important antioxidant activity, together with gallic acid. Tannins also appear to be responsible for astringent and hemostatic properties and enhance tissue regeneration [155,156].

The anti-inflammatory activity of *Hamamelis virginiana* L. appears to result from the vasoconstrictor activity of tannins as well as from the inhibition of histamine release by flavonoids, which together decrease skin blood flow. These effects justify the improvement of dermatitis when topically applied to healthy subjects [156,157].

In addition to antioxidant activity, the extract of *Hamamelis virginiana* L. also shows an inhibitory effect on collagenase, elastase, and alpha-glucosidase, contributing to the stabilization of the vascular wall and ameliorating the symptoms of venous diseases [152,153].

*Hamamelis virginiana* L. is generally well tolerated, with known sporadic adverse effects including gastric irritation and dermatitis. Due to lack of data, it is not advised to be administered during pregnancy [158].

### 4.7. Vitis vinifera *L.*

**Figure 9:**
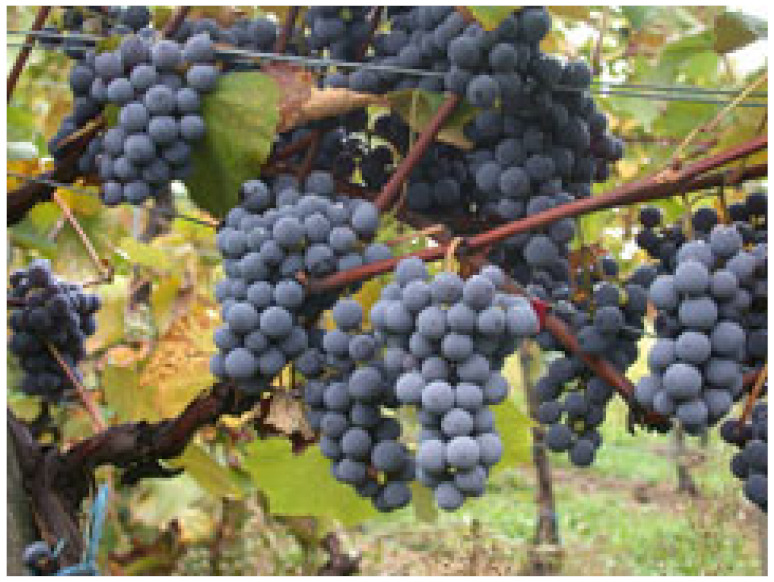
*Vitis vinifera* L. photo.

*Vitis vinifera* L. (Vitaceae) (Figure 9) or common grapevine is indigenous to southern Europe and Western Asia, and is the most widely cultivated and economically important fruit crop in the world [159]. It has been used for millennia for its nutritional and medicinal properties. The main bioactive compounds of the leaves of *Vitis vinifera* L. are phenolic compounds, such as stilbenoids (resveratrol), phenolic acids (gallic acid), flavan-3-ols (catechin), flavonols, procyanidins, anthocyanins, and leucocyanidines. Its seeds are also a source of polyphenols, such as procyanidins, flavonoids, and catechins [160]. It has traditionally been used in the treatment of capillary bleeding, edema, and inflammation, for example, in neurological problems derived from microvascular dysfunction [160]. Resveratrol and procyanidins are beneficial in preventing microvascular disease [161,162], namely by their endothelial-protecting, vasorelaxing, and antiangiogenic activities.

*Vitis vinifera* L. extract protects the endothelium against oxidative stress in vitro [163]. Additionally, it potentiates endothelial-dependent vasorelaxation, probably by increasing NO synthesis [164]. In animals, an extract of unripe grape is able to decrease blood pressure in response to the administration of angiotensin II [165]. Furthermore, in several animal models, *Vitis vinifera* L. extract is able to decrease vascular permeability due to its anti-inflammatory activity, namely the inhibition of NO and prostaglandins by leucocytes, which is attributed to the suppression of the NF-κB pathway [166]. This anti-inflammatory activity justifies the clinical improvement of patients with PVD, namely skin perfusion [167]. Finally, *Vitis vinifera* L. has antiangiogenic activity, as it inhibits the secretion of the pro-angiogenesis factor VEGF in vitro [162,168].

Adverse effects caused by the administration of *Vitis vinifera* L. extracts are mostly related to the gastrointestinal tract, such as nausea, anorexia, headaches, and allergic manifestations. Otherwise, it is considered to be generally well tolerated [169].

### 4.8. Ginkgo biloba *L.*

**Figure 10:**
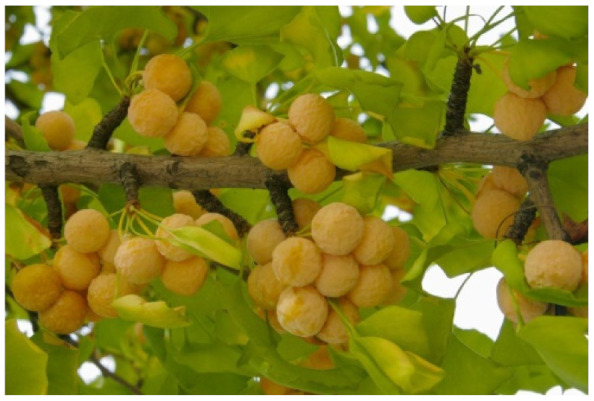
*Ginkgo biloba* L. photo.

*Ginkgo biloba* L. (Figure 10) is the oldest tree in the world and has gone unchanged for approximately 270 million years. It has no living relative in existence, which is why it has its own division, Ginkgophyta. Gingko leaves have been used in traditional Chinese medicine for hundreds of years, and are the source of a currently-made standardized extract that is among the top five best-selling herbal products. Its leaves contain terpene trilactones (ginkgolides and bilobalides) and flavonoids (quercetin and kaempferol), among other compounds [170].

There are many publications on the beneficial uses of *Ginkgo biloba* L. for cerebrovascular disease and related neurologic conditions such as vascular dementia. In microvascular terms, it has several beneficial effects, namely its vasodilator and endothelial-protecting activities.

Regarding its endothelium-protecting activities, *Ginkgo biloba* L. protects against oxidative stress induced by oxidized low-density lipoproteins [171] by inhibiting the expression of inducible nitric oxide synthase (iNOS) [172]. It also decreases the senescence of endothelial-progenitor cells by increasing telomerase activity [173]. It inhibits the endothelial expression of intercellular adhesion molecule 1 (ICAM-1) and vascular cell adhesion molecule 1 (VCAM-1) in vitro, which suggests its usefulness for preventing the adhesion of leucocytes [174].

Regarding its vasodilator activity, supplementation with *Ginkgo biloba* L. has been shown to increase perfusion in several vascular beds, namely retinal, cochlear, cerebral, and coronary, although conflicting results have been reported for cutaneous microcirculation. *Ginkgo biloba* L. supplementation increases nailfold [175] and forearm [176] perfusion in healthy subjects. However, in the forefoot, an extract of *Ginkgo biloba* L. evoked different responses after 3 weeks of supplementation (240 mg/day) [177]. In retinal microcirculation, no effect was observed in healthy subjects after 3 h of oral administration of 240 mg of extract 761 (EGb 761) [178]. However, a similar study reported that in healthy subjects, taking 40 mg of extract thrice daily led to a significant increase in retinal perfusion after 1 week and up to 4 weeks [179] in patients with normal tension glaucoma [180], and retinal perfusion increased in diabetic retinopathy [181]. Cochlear microcirculation also seems to benefit from *Ginkgo biloba* L., in part due to its effect in ameliorating hemorheology [182]. In a guinea pig model, extract EGb 761 protected cochlear microcirculation against stressful stimuli (hypoxia, salicylate, lipopolysaccharide) [183,184] by preventing damage to hair cells and, consequently, cochlear and vestibular dysfunction. However, these positive effects are yet to be shown in human subjects. *Ginkgo biloba* L. extract also increases cerebral perfusion in both animals [185] and humans [186,187]. In a small pilot study with healthy elderly males, cerebral perfusion significantly increased, particularly in the left parietal-occipital region, after taking a 60 mg extract for 4 weeks [184]. In patients with the vascular cognitive impairment of non-dementia who took a *Ginkgo biloba* L. extract of 19.2 mg thrice daily for 3 months, together with standard antiplatelet medication, anterior cerebral artery perfusion significantly increased, as did cognitive function test scores [188]. Finally, in healthy subjects, an extract of *Ginkgo biloba* L. was administered intravenously (0.7 mg/min) for 120 min, during which time coronary perfusion significantly increased [189]. This vasodilator activity seems to be attributed to the potentiation of endothelium-dependent vasodilation [189], the suppressing effect on the synthesis of endothelin-1 (ET-1) [190], and vasomotion regulation.

The vasodilator and endothelium-potentiation effects, together with angiotensin-converting enzyme inhibition, explain the blood pressure lowering effect in spontaneously hypertensive and 2K1C hypertensive rats [191,192]. However, *Ginkgo biloba* L. does not seem to be potent enough to lower blood pressure in humans, at least not in elderly subjects [193].

Some sporadic adverse responses have been linked with hemorrhagic complications [194,195], including one example of subdural hematoma [196]. The activity is most likely due to the ginkgosides’ antiplatelet activity, with ginkgolide B appearing to be the major terpenoid involved [196,197]. Other research works report other symptoms as follows: acute generalized exanthematous pustulosis [198]; toxic epidermal necrolysis [199]; ventricular arrhythmia [200]; and convulsions [201]. An increased risk of bleeding complications has been observed when *Ginkgo biloba* L. was taken concomitantly with other conventional drugs that act on coagulation, such as acetyl salicylic acid [202,203], ibuprofen [204], and warfarin [205]. In a case of fatal breakthrough seizure, a subtherapeutic level of anticonvulsants (phenytoin and valproic acid) has also been reported owing to an increase in CYP2C19 by ginkgo active substances [206].

### 4.9. Salvia miltiorrhiza Bunge

**Figure 11:**
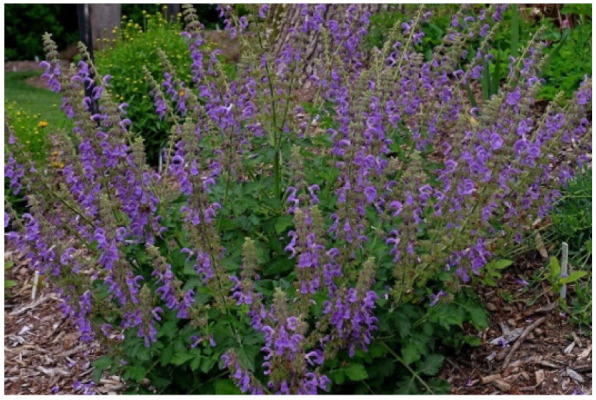
*Salvia miltiorrhiza* L. photo.

*Salvia miltiorrhiza* Bunge (Lamiaceae), also known as “Danshen” in China (Figure 11), is an aromatic perennial herb distributed in China and Japan. The roots, rhizomes, stems, and leaves of *Salvia miltiorrhiza* Bunge have been used in traditional Chinese medicine to treat numerous diseases, especially CVD [207]. The principal bioactive components in this herb are diterpenoids, namely tanshinones, and phenolic acids such as salvianolic acids [208]. *Salvia miltiorrhiza* Bunge plays a beneficial role in microcirculation by protecting the endothelium due to its vasodilator and anti-inflammatory activities and to its modulation ability in angiogenesis.

Regarding the protective role of the endothelium, *Salvia miltiorrhiza* Bunge has been shown to protect against not only oxidative stress [209], but also homocysteinemia-induced endothelial dysfunction in vitro [210]. Finally, this herb is also able to reduce the endothelial permeability evoked by TNFα [211] and to inhibit the endothelial adhesion of leucocytes [212].

The vasodilator activity of *Salvia miltiorrhiza* Bunge seems to be attributed to its ability to suppress the expression of thromboxane-A_2_ and ET-1, as observed in an animal model of dextran-induced microvascular dysfunction, and therefore, counteracts their vasoconstrictor effects [213]. In a model of ovarectomized rats fed a high-fat diet, *Salvia miltiorrhiza* Bunge was also able to upregulate eNOS expression and to increase NO secretion [214]. This vasodilator activity together with antiplatelet and anti-inflammatory effects, namely the downregulation of adhesion molecules and the suppression of cytokine secretion, contribute to the beneficial effects of *Salvia miltiorrhiza* Bunge extracts on cerebral I/R injury [215].

With its wide variety of uses, *Salvia miltiorrhiza* Bunge products have been linked with a growing variety of adverse effects, including abdominal discomfort, convulsions, dystonia syndrome [216], reduced appetite [217], and allergies. These effects subside when intake is discontinued. In animals, a high dose of intravenously administered *Salvia miltiorrhiza* Bunge causes vascular toxicity with increasing ET-1 levels [217].

### 4.10. Mangifera indica *L.*

**Figure 12:**
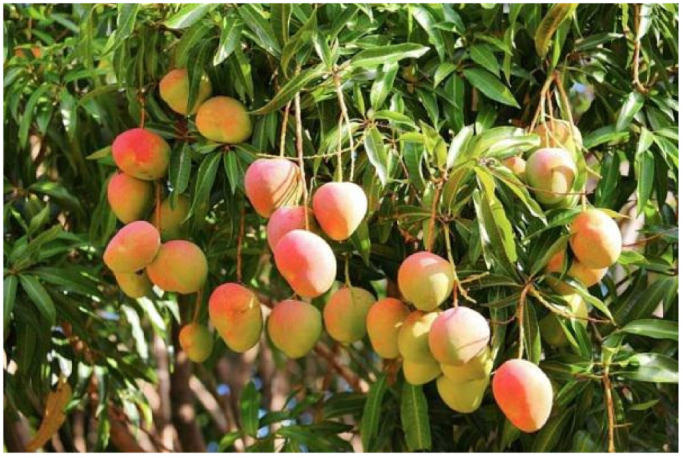
*Mangifera indica* L. photo.

The fruit and leaves of *Mangifera indica* L. (Anacardiaceae), commonly known as Mango (Figure 12), are rich in polyphenols, including the xanthonoid mangiferin, flavonoids (e.g., procyanidins), hydroxybenzoic (e.g., gallic, vanillic, syringic, protocatechuic, and *p*-hydroxybenzoic acids), and hydroxycinnamic (e.g., *p*-coumaric, chlorogenic, ferulic, and caffeic acids) acid derivatives [218,219].

In a human pilot study, a powder supplement containing 100% of the *Mangifera indica* L. fruit improved microcirculation by potentiating the reactive hyperemia response, an effect which was attributed to the increase in eNOS expression, as observed in the endothelial cells in vitro [220]. Another study showed that the same extract was able to improve the reactive hyperemia evoked in the postprandial state after a high glucose intake, a factor known to impair the endothelium [221]. To our knowledge, no adverse effects related to the *Mangifera indica* intake have been reported.

## 5. Conclusions

Despite the limited scientific evidence for the influence of diet on microcirculation, as far as the authors know, this is the first paper to provide a comprehensive review with considerations on both previous and the most recent literature centering on the impact that food supplementation may play in microcirculation. As there is likely to be an increase in the use of food supplements to improve personal health, developing a global consensus about their regulations before they are marketed is essential. We have not accounted for any cultural and legal differences that exist in different countries when products are developed as food supplements, which is a limitation.

In addition to further individualized knowledge about the sources referred to in Section 4, which can serve as a basis for the formulation of various food supplements that act at the microcirculation level, it is important to further investigate the combination of one or more of these sources to formulate food supplements to obtain a clinically proven impact. In addition, further clinical trials are necessary to better explore the effects of these food supplements on microcirculation.

## Figures and Tables

**Table 1:** List of authorized claims in the USA with direct or indirect impact on microcirculation.

Authorized Health Claims	Qualified Health Claims
Dietary saturated fat and cholesterol and risk of coronary heart disease	Whole grain foods with moderate fat content and risk of heart disease
Fruit, vegetables, and grain products that contain fiber, particularly soluble fiber, and risk of coronary heart disease	Saturated fat, cholesterol, and trans fat, and reduced risk of heart disease
Soluble fiber from certain foods and risk of coronary heart disease	Substitution of saturated fat in diet for unsaturated fatty acids and reduced risk of heart disease
Soy protein and risk of coronary heart disease	B vitamins and vascular disease
Plant sterol/stanol esters and risk of coronary heart disease	Nuts and heart disease
	Walnuts and heart disease
	Omega 3 fatty acids and coronary heart disease
	Monounsaturated fatty acids from olive oil and coronary heart disease
	Unsaturated fatty acids from canola oil and reduced risk of coronary heart disease
	Corn oil and corn oil-containing products and a reduced risk of heart disease

**Table 2:** List of nonauthorized claims in the EU with the word microcirculation.

Nutrient, Substance, Food, or Food Category	Claim	Ref
Dry isoflavones soy Extract	Acts on hair bulb to support hair growth. Prevents hair from premature aging via antioxidant properties and microcirculation.	[56]
Vitamin B3	Activates scalp microcirculation.	[57]
Bioflavonoids	It has a positive effect on microcirculatory tropism by favoring the processes that protect small venous vessels. It protects the body from the harmful action of free radicals and skin from ultraviolet rays.	[58]
Vitamin E acetate (D,L alpha tocopherol acetate)	It supports microcirculation and scalp oxygenation.	[59]
OPC Plus, containing 40 mg oligomeric procyanidins (OPC) and 40 mg berry blend per capsule	OPC Plus has been shown to increase microcirculation and may, therefore, reduce the risk of chronic venous insufficiency.	[60]

## Data Availability

Not applicable.
