# Correction: Raposo et al. The Role of Food Supplementation in Microcirculation—A Comprehensive Review. *Biology* 2021, *10*, 616

**DOI:** 10.3390/biology12091198

**Published:** 2023-09-01

**Authors:** António Raposo, Ariana Saraiva, Fernando Ramos, Conrado Carrascosa, Dele Raheem, Rita Bárbara, Henrique Silva

**Affiliations:** 1 CBIOS (Research Center for Biosciences and Health Technologies), Universidade Lusófona de Humanidades e Tecnologias, Campo Grande 376, 1749-024 Lisboa, Portugal; 2 Department of Animal Pathology and Production, Bromatology and Food Technology, Faculty of Veterinary, Universidad de Las Palmas de Gran Canaria, Trasmontaña s/n, 35413 Arucas, Spain; ariana_23@outlook.pt (A.S.); conrado.carrascosa@ulpgc.es (C.C.); 3 Pharmacy Faculty, University of Coimbra, Azinhaga de Santa Comba, 3000-548 Coimbra, Portugal; framos@ff.uc.pt; 4 REQUIMTE/LAQV, Rua Dom Manuel II, Apartado 55142, 4051-401 Oporto, Portugal; 5 Northern Institute for Environmental and Minority Law (NIEM), Arctic Centre, University of Lapland, 96101 Rovaniemi, Finland; braheem@ulapland.fi; 6 School of Sciences and Health Technologies, Universidade Lusófona de Humanidades e Tecnologias, Av. Campo Grande 376, 1749-024 Lisbon, Portugal; a21705573@alunos.ulht.pt; 7 Research Institute for Medicines (iMed.ULisboa), Faculdade de Farmácia, Universidade de Lisboa, Av. Prof. Gama Pinto, 1649-003 Lisbon, Portugal; 8 Department of Pharmacy, Pharmacology and Health Technologies, Faculdade de Farmácia, Universidade de Lisboa, Av. Prof. Gama Pinto, 1649-003 Lisbon, Portugal

Following the publication of [1], a number of citation issues were found to have occurred due to an error in the references management tool used. These errors resulted in a number of significant citations being omitted from the text and reference section, a number of citations being duplicated and issues with the order and numbering of the citations with the text and reference section. As such, changes to the text, tables and figures related to these citation errors have been made. A number of other minor typographic and formatting errors have also been corrected. 

The original paper has been updated as follows:

## 1. Text Corrections

On page 4, Section 2, fourth paragraph, line 3, the sentence “(French Food Safety Agency (AFSSA, Agence Française de Sécurité Sanitaire des Aliments); Bundesinstitut für Risikobewetung (BFR), (Germany), 2018; (Spanish Agency for Food Safety and Nutrition (AESAN), 2018).” Has been be changed to “(French Food Safety Agency (AFSSA, Agence Française de Sécurité Sanitaire des Aliments); Food Standards Agency (UK), 2018; Bundesinstitut für Risiko-bewetung (BFR), (Germany), 2018; Spanish Agency for Food Safety and Nutrition (AESAN), 2018 [33,35,36,37]”.

On page 4, Section 2, fifth paragraph, line 1, the word “the” should be added after “…is linked with”.

On page 4, Section 2, fifth paragraph, line 9, the word “Hachen et al.” should be changed to “Hachem et al.”.

On page 7, Section 2, third paragraph, line 2, the sentence “The lack of safety insight has been counteracted by numerous studies showing different results concerning the positive effects of food supplements and quality products traded in Europe, including metal in preparations of spirulina tablets [38]; vitamin D [39]; ginseng herbal medicine control and authentication [40]; cottonseed oil and cottonseed meal supplementation [41]; supplemental lycopene on cardiovascular risk factor [42]; and propolis as an antioxidant and antimicrobial [43]”. Has be updated to “The lack of safety insight has been counteracted by numerous studies showing different results concerning the positive effects of food supplements and quality products traded in Europe, including testosterone-boosting supplements (T-Boosters) [45], metal in preparations of spirulina tablets [46], vitamin D [47], ginseng herbal medicine control and authentication [48], cottonseed oil and cottonseed meal supplementation [49], supplemental lycopene on the cardiovascular risk factor [50], and propolis as an antioxidant and antimicrobial agent [51]”.

On page 8, Section 3, sixth paragraph, line 4, the word “given EFSA’s...” should be changed to “given the EFSA’s...”.

On page 9, Section 3, seventh paragraph, line 2, the word “depending on dose” should be changed to “depending on the dose...”.

On page 9, Section 4.1, first paragraph, line 4, the word “identified in...” should be updated to “identified in the...”.

On page 10, Section 4.1, fourth paragraph, line 2, the word “increase...” should be updated to “increase the...”.

On page 11, Section 4.3, fourth paragraph, line 4, the word “occur in...” should be updated to “occur in the...”.

## 2. References Section Changes

The following references have been added to the Reference section:

28. Petroczi, A.; Taylor, G.; Naughton, D.P. Mission impossible? Regulatory and enforcement issues to ensure safety of dietary supplements. *Food Chem. Toxicol.* **2011**, *49*, 393–402.
29. Mannino, G.; Di Stefano, V.; Lauria, A.; Pitonzo, R.; Gentile, C. Vaccinium macrocarpon (Cranberry)-based dietary supplements: Variation in mass uniformity, proanthocyanidin dosage and anthocyanin profile demonstrates quality control standard needed. *Nutrients* **2020**, *12*, 992.
30. European Commission. Regulation (EC) No 178/2002 of the European Parliament and of the Council of 28 January 2002 laying down the general principles and requirements of food law, establishing the European Food Safety Authority and laying down procedures in matters of food safety. *Off. J. Eur. Comm.* **2002**, *L31*, 1–24.
31. European Commission. Directive 2002/46/EC of the European Parliament and of the Council of 10 june 2002 on the approximation of the laws of the member states relating to food supplements. *Off. J. Eur. Union* **2000**, *L183*, 51–57.
32. Cerezo, A.B.; Leal, Á.; Álvarez-Fernández, M.A.; Hornedo-Ortega, R.; Troncoso, A.M.; García-Parrilla, M.C. Quality control and determination of melaonin in food supplements. *J. Food Compos. Anal.* **2016**, *45*, 80–86.
33. Agencia Española de Consumo y Seguridad Alimentaria y Nutrición (AECOSAN). Ministerio de Sanidad y Consumo de España. Programa 10. Control de Complementos Alimenticios: Notificación, Etiquetado y Composición. 2018. Informe Anual 2019. Available online: https://www.aesan.gob.es/AECOSAN/docs/documentos/seguridad_alimentaria/pncoca/P10_Complementos.pdf (accessed on 25 January 2021).
34. Fibigr, J.; Šatínský, D.; Solich, P. Current trends in the analysis and quality control of food supplements based on plant extracts. *Anal. Chim. Acta* **2018**, *1036*, 1–15.
35. French Food Safety Agency. Food Supplements, the Need for Informed Consumption. 2021. Available online: https://www.anses.fr/en/content/food-supplements-need-informed-consumption (accessed on 25 January 2021).
36. Food Standards Agency (UK). Food Supplements. What Food Supplements Are and What You Need to Do as a Business to Sell Them. 2018. Available online: https://www.food.gov.uk/business-guidance/food-supplements (accessed on 25 January 2021).
37. BFR, Frequently Asked Questions on Food Supplements. 2018. Available online: https://www.bfr.bund.de/en/frequently_asked_questions_on_food_supplements-70347.html (accessed on 25 January 2021).
40. Sanzini, E.; Badea, M.; Dos Santos, A.; Restani, P.; Sievers, H. Quality control of plant food supplements. *Food Funct.* **2011**, *2*, 740–746.
41. European Commission. RASFF—Food and Feed Safety Alerts. 2021. Available online: https://ec.europa.eu/food/safety/rasff_en (accessed on 25 January 2021).
43. Gershwin, M.E.; Borchers, A.T.; Keen, C.L.; Hendler, S.; Hagie, F.; Greenwood, M.R.C. Public safety and dietary supplementation. *Ann. N. Y. Acad. Sci.* **2010**, *1190*, 104–117.
44. Cassileth, B.R.; Heitzer, M.; Wesa, K. The public health impact of herbs and nutritional supplements. *Pharm. Biol.* **2009**, *47*, 761–767.
45. Balasubramanian, A.; Thirumavalavan, N.; Srivatsav, A.; Yu, J.; Lipshultz, L.I.; Pastuszak, A.W. Testosterone imposters: an analysis of popular online testosterone boosting supplements. *J. Sex. Med.* **2019**, *16*, 203–212.
46. Rubio, C.; Dominik-Jakubiec, M.; Paz, S.; Gutiérrez, Á.J.; González-Weller, D.; Hardisson, A. Dietary exposure to trace elements (B, Ba, Li, Ni, Sr, and V) and toxic metals (Al, Cd, and Pb) from the consumption of commercial preparations of Spirulina platensis. *Environ. Sci. Pollut. Res.* **2021**, *28*, 22146–22155.
47. Starek, M.; Mierzwa, J.; Gumułka, P.; Dąbrowska, M. Vitamin D–current stage of knowledge about analysis and supplementation. *Crit. Rev. Food Sci. Nutr.* **2022**, *62*, 4607–4621.
48. Ichim, M.C.; de Boer, H.J. A Review of Authenticity and Authentication of Commercial Ginseng Herbal Medicines and Food Supplements. *Front. Pharmacol.* **2020**, *11*, 612071.
49. Yang, A.; Zhang, C.; Zhang, B.; Wang, Z.; Zhu, L.; Mu, Y.; Qi, D. Effects of Dietary Cottonseed Oil and Cottonseed Meal Supplementation on Liver Lipid Content, Fatty Acid Profile and Hepatic Function in Laying Hens. *Animals* **2021**, *11*, 78.
50. Tierney, A.C.; Rumble, C.E.; Billings, L.M.; George, E.S. Effect of dietary and supplemental lycopene on cardiovascular risk factors: A systematic review and meta-analysis. *Adv. Nutr.* **2020**, *11*, 1453–1488.
51. Ucak, I.; Khalily, R.; Carrillo, C.; Tomasevic, I.; Barba, F.J. Potential of Propolis Extract as a Natural Antioxidant and Antimicrobial in Gelatin Films Applied to Rainbow Trout (*Oncorhynchus mykiss*) Fillets. *Foods* **2020**, *9*, 1584.
53. EFSA (European Food Safety Authority), 2018. Outcome of a Public Consultation on the Draft Guidance for the Scientific Requirements for Health Claims Related to Antioxidants, Oxidative Damage and Cardiovascular Health. EFSA Supporting Publication 2018:EN-1364. p. 35. Available online: https://efsa.onlinelibrary.wiley.com/doi/abs/10.2903/sp.efsa.2018.EN-1364 (accessed on 25 January 2021).
68. Rogerson, D.; Maçãs, D.; Milner, M.; Liu, Y.; Klonizakis, M. Contrasting effects of short-term Mediterranean and vegan diets on microvascular function and cholesterol in younger adults: A comparative pilot study. *Nutrients* **2018**, *10*, 1897.

The following references have been removed from the Reference section:

27. Dzeparoski, M.; Trajkovic-Jolevska, S. Analysis of marketing strategy for food supplements and over the-counter medicines. *Maced. J. Med. Sci.* **2016**, *4*, 499–503.
33. Public Law 101–535—Nov. 8, 1990. Nutrition Labeling and Education Act of 1990. Available online: https://www.congress.gov/101/statute/STATUTE-104/STATUTE-104-Pg2353.pdf (accessed on 20 January 2021).
34. FDA. Label Claims for Conventional Foods and Dietary Supplements 2019. Available online: https://www.fda.gov/food/foodlabeling-nutrition/label-claims-conventional-foods-and-dietary-supplements (accessed on 25 January 2021).

All other reference numbers have updated accordingly.

The authors state that the scientific conclusions are unaffected. This correction was approved by the Academic Editor. The original publication has also been updated.
